# Cardiovascular abnormalities in patients with oral cleft: a clinical-electrocardiographic-echocardiographic study

**DOI:** 10.6061/clinics/2018/e108

**Published:** 2018-04-09

**Authors:** Gisele C.P. Leite, Marcela A.G. Ururahy, João F. Bezerra, Valéria M.G.D.M. Lima, Maria I.F. Costa, Sandra S.C. Freire, André D. Luchessi, Jussara M.C. Maia, Maria E.F. Brito, Vera L. Gil-da-Silva-Lopes, Adriana A. Rezende

**Affiliations:** I Departamento de Pediatria, Universidade Federal do Rio Grande do Norte, Natal, RN, BR; II Departamento de Analises Clinicas e Toxicologicas, Universidade Federal do Rio Grande do Norte, Natal, RN, BR; III Departamento de Genetica Medica, Universidade Estadual de Campinas, Campinas, SP, BR

**Keywords:** Oral Cleft, Congenital Heart Disease, Rheumatic Heart Disease, Mitral Valve Prolapse

## Abstract

**OBJECTIVES:**

The present study aims to describe the clinical, electrocardiographic, and echocardiographic cardiological findings in a group of patients with oral clefts.

**METHODS:**

This is a prospective cross-sectional study on 70 children (age range from 13 days to 19 years) with oral clefts who attended the multidisciplinary program of a university hospital from March 2013 to September 2014. The patients were evaluated by a pediatric cardiologist and underwent detailed anamnesis, physical examination, electrocardiogram, and echocardiogram.

**RESULTS:**

Sixty percent of the patients were male; 55.7% presented with cleft lip and palate, and 40.0% presented with health complaints. Comorbidities were found in 44.3%. Relevant pregnancy, neonatal, family and personal antecedents were present in 55.7%, 27.1%, 67.2%, and 24.3% of the patients, respectively. Regarding the antecedents, 15.2% of the patients presented with a cardiac murmur, 49.0% with a familial risk of developing plurimetabolic syndrome, and 6% with family antecedents of rheumatic fever. Electrocardiographic evaluation showed one case of atrioventricular block. Echocardiograms were abnormal in 35.7% of the exams, including 5 cases of mitral valve prolapse — one of which was diagnosed with rheumatic heart disease.

**CONCLUSION:**

The finding of a family risk of developing plurimetabolic syndrome and a diagnosis of rheumatic heart disease indicates that patients with oral clefts may be more prone to developing acquired heart disease. Thus, our findings highlight the importance of anamnesis and methodological triangulation (clinical-electrocardiographic-echocardiographic) in the investigation of patients with oral clefts and emphasize that cardiological follow-up to evaluate acquired and/or rhythm heart diseases is necessary. This strategy permits comorbidity prevention and individualized planned treatment.

## INTRODUCTION

Oral clefts (OCs) are a heterogeneous group of important congenital defects with a prevalence of 1:500-1000 live births 1,2. OCs are recognized by the World Health Organization (WHO) as a public health problem 3. In 70% of cases, OCs have an isolated presentation (non-syndromic), whereas the rest are associated with other congenital defects (syndromic) 1,2. According to Fogh-Andersen 4, OCs are classified as cleft lip (CL), cleft palate (CP), and cleft lip and palate (CLP).

The incidence of malformations associated with OCs varies widely in the literature, ranging from 1.5% to 63% 5. These associations are extremely important for clinical follow-up and appropriate multidisciplinary management 6.

Cardiovascular malformations are one of the most common congenital anomalies in patients with CLP 5. Congenital heart disease (CHD) has been reported as the most common anomaly associated with OC in Jordan 7, Pakistan 8, and China 5.

CHD prevalence is higher in children with OC than in the general pediatric population and occurs at various rates (5.4%-15%) in different studies 9,10. These CHDs include atrial septal defect (ASD), ventricular septal defect (VSD), patent foramen ovale (PFO), patent ductus arteriosus (PDA), Tetralogy of Fallot (TOF), truncus arteriosus, transposition of the great vessels, and pulmonary hypertension (PH) 10-12. Studies regarding cardiac defects in children with OC are usually anatomic and include echocardiographic findings 5,8,10-12, with a few studies correlating with clinical and echocardiographic findings 7,9,13-15. To the best of our knowledge, there is no study describing and correlating clinical, electrocardiographic, and echocardiographic findings with cardiac rhythm abnormalities, CHD, and/or AHD.

Thus, this study aims to describe the findings of a clinical-electrocardiographic-echocardiographic cardiological evaluation in a sample of individuals with OCs.

## PATIENTS AND METHODS

This is a prospective cross-sectional study on patients with OCs who attended the Multidisciplinary Program for Patients with CL and/or CP at University Hospital Onofre Lopes (HUOL)/Federal University of Rio Grande do Norte (UFRN), Natal/RN, Brazil, from March 2013 to September 2014. OCs were categorized into CL, CP, and CLP according to the Fogh-Andersen classification 4.

From the total 96 recruited patients, 70 were evaluated by the same pediatric cardiologist (GCPL) and underwent detailed anamnesis, physical examination, electrocardiogram (ECG) and echocardiogram. The evaluated variables were age, sex, OC type, clinical evidence of heart disease probability (pregnancy, neonatal, family and personal antecedents) and electrocardiographic and echocardiographic findings. The echocardiogram was performed using a Philips iE33 (Philips Medical Systems, Andover, Massachusetts, USA), and the ECG was performed using a DIXTAL model EP-3 (DIXTAL Biomédica Indústria e Comércio Ltda, Brazil). Cardiovascular malformations were defined according to the American Heart Association 16. The severity of CHD was described according to Hoffman and Kaplan 17 as mild, moderate, or severe.

Statistical analyses were performed using the SPSS software, version 21.0 (IBM Inc., USA). The results are expressed in tables of frequencies and percentages.

This study is part of Brazil’s CranioFacial Project (http://www.fcm.unicamp.br/fcm/cranio-face-brasil/projeto-cranio-face-brasil) 18 and was approved by the Ethics and Research Committee of UFRN. All who were legally responsible for the participants signed a written consent form.

### Ethics

This study was carried out according to the ethical standards of the committee responsible for human experimentation (institutional or regional) and the Helsinki Declaration of 1975, revised in 1983, according to the Institution’s Ethics and Research Committee numbers (CAAE: 30958114.0000.5292).

## RESULTS

### Patients

Table 1 shows the clinical and diagnostic data of patients with OCs at the time of the pediatric cardiologist evaluation. Of the 70 patients, 42 (60.0%) were male. Patient ages ranged from 13 days to 19 years old (mean: 4.64 years). Ten (14.3%) patients were younger than a year old (one neonate and nine with ages ranging from 2 to 10 months).

Regarding OC type, 40 (55.1%) patients presented with CLP, and familial cleft history was noted in 32.9% of patients. A pediatrician evaluated all patients, and most had undergone some type of OC correction. Forty-five (64.3%) were evaluated by a geneticist, with two (2.9%) cases of diagnosed syndromes and nine (12.9%) cases suspected of having a syndrome.

Regarding the clinical evaluation, 28 (40.0%) patients had general health complaints. Thirty-one (44.3%) patients presented with comorbidities (Table 2).

### Cardiovascular evaluation

Table 3 shows the pregnancy (PrA), neonatal (NA), family (FA), and personal antecedents (PeA).

Among the nine (11.8%) pregnancies with malformations on obstetric ultrasound, four had OC diagnosis, and one was a suspected case of fetal cardiac malformation. This patient had to undergo surgical treatment of PDA evolving with PH.

FA was found in 47 (67.2%) patients, and six (6.0%) cases had rheumatic fever (RF). From the 10 FA of heart diseases, five were acquired (one case of RHD), and five were congenital. Regarding the congenital cases, one was a cousin with a cardiac murmur and a history of sudden death at 21 years old, and another was a cousin with a diagnosis of Down syndrome. Of the six cases with FA of RF, two were the patients’ mothers, and one was an aunt with RHD who underwent cardiac surgery at 35 years of age.

Relevant PeA was present in 17 individuals (24.3%). Heart diseases occurred in two (8.7%). One presented with ASD noted on echocardiogram on the 5^th^ day of life, which closed spontaneously during the 7^th^ month of life, but the patient developed extrasystoles after the first year. The other patient presented with agenesis of the corpus callosum, PDA and PH by hyperflow and underwent surgical treatment of PDA, but the patient had persistent PH and was noted to have a large ASD on subsequent echocardiography.

In regard to ECG (Table 4), 16 (22.9%) patients were found to have abnormalities upon final evaluation, with 14 (87.6%) cases of right bundle branch block (RBBB). A patient who underwent cardiological preoperative evaluation for OC correction presented with atrioventricular block (AVB) on ECG and is presently under investigation.

Echocardiography was performed in all patients, and 25 (35.7%) abnormalities were found in the examinations (Table 5). Of these, 18 patients had isolated abnormalities in the echocardiogram (10 VSDs, 5 mitral valve prolapses or MVPs, 2 PFOs, and 1 PDA), whereas seven had more than one alteration. In two cases, there was suspicion of left ventricular noncompaction (LVNC): one had arthrogryposis syndrome and died before the cardiac angiotomography (CT) was performed; the other underwent CT, and heart disease was ruled out.

Of the two syndromic patients, the one with Noonan syndrome presented with normal ECG but with a PFO on echocardiography. The aforementioned patient with arthrogryposis presented with RBBB and diffused alteration of ventricular repolarization in the ECG and dilatation of the ascending aorta, suspicion of LVNC and endomyocardial fibrosis in the right ventricle on echocardiogram. This patient died without undergoing CT.

One patient had a history of recurrent tonsillitis and hip joint pain, which was confirmed to be RHD with MVP. Appropriate treatment and prophylaxis for bacterial endocarditis were implemented, leading to improvement in the mitral valve injury and cure of arthralgia at follow-up.

Regarding the severity of heart disease, 92% were classified as mild, 4% as moderate, and 4% as severe.

## DISCUSSION

OCs are considered to be a public health problem by the WHO because of their prevalence and the need for integrated, long-term specialized and multidisciplinary treatment 3. In this context, the present study aimed to characterize individuals with OCs based on routine investigation with methodological triangulation (clinical-electrocardiographic-echocardiographic findings). As a differential, non-anatomic variables were also included to evidence various risks that may influence global individual treatments. Notably, there was no separation in syndromic and non-syndromic OC; this strategy was adopted to widely characterize the cardiological findings.

The higher frequency of male patients with OCs was similar to that in some literature reports 2,10,12; however, other studies 6,13 showed a uniform distribution of OCs in both sexes.

This study showed that among the types of OCs, CLP had the highest frequency, followed by CP and CL, which corroborated the findings of Baptista 6. However, other studies found a higher prevalence of CP 3,9, and another report showed similar frequencies for all three types 5. Available literature regarding the correlation between OC type and associated malformations remains controversial worldwide 5,19, perhaps stemming from variations in study type, methods, and evaluated populations.

In the present study, 32.9% of cases had FA of OC, which was in accordance with the results of Baptista 6, and 23.0% of these cases were found to have family histories, reinforcing the hereditary nature of this malformation. Shafi 8 found family histories of OC in 23% of children with associated anomalies and in 22% of children without associated anomalies. Genetic susceptibility has been identified as a major component of CLP 19-21.

Most patients with OC do not present with any other abnormality (non-syndromic OC); however, a significant portion of patients (30-50%) still present with other malformations that may be related to an unknown syndrome due to the difficult access to genetic consultation and examinations 22. This scenario is common in Brazil, where access to genetic services is limited 18,23,24. Sun et al. 5 found that 30.1% of OC patients had other congenital anomalies, whereas Wyse et al. 11 found abnormalities in other systems in 87% of patients with OCs and CHD. According to Baptista 6, diagnosing these malformations is important for appropriate clinical follow-up and genetic counseling, even when the specific syndromic diagnosis is not conclusive.

The frequency of complaints related to cardiac abnormalities reinforced the need for a cardiological check-up in patients with OCs, as reported by Harry 9.

The recurrent upper airway infections and hearing impairment found in this population may be related to anatomic craniofacial abnormalities 6. The other described comorbidities may be associated with the combined occurrence of OC with other anomalies, and they need to be determined to improve clinical and therapeutic follow-up in these patients.

Cardiac anomalies may be isolated (80-85%) or part of chromosomal (5-10%) or genetic (3-5%) syndromes 25. The heart and palate may develop abnormally as a result of genetic and environmental factors during embryogenesis 9. Several genes have been identified in syndromes that affect the heart and palate; however, the fundamental molecular mechanisms of non-syndromic OCs remain relatively unexplored 9.

The findings of positive PrA, NA, FA, and PeA in a significant portion of the patients reinforce the importance of genetic and environmental factors in both anomalies (OC and cardiovascular malformations). Notably, 15.2% of the patients presented with a cardiac murmur, 49.0% presented with a familial risk of developing plurimetabolic syndrome (arterial hypertension – 20.0%, dyslipidemia – 17.0%, and diabetes mellitus – 12.0%), and 6% had an FA of RF. These patients have OCs that showed abnormalities in 35.7% of the echocardiographic findings. This result reinforces the importance of a cardiologic exam in the evaluation and follow-up of these patients, including their preoperative evaluation 6,7,10,13, to screen for both congenital and acquired cardiovascular abnormalities; thus, cardiac risk evaluation in OC patients must also address the prevention of AHD.

The described PrA revealed risk factors for malformations in general, such as maternal diseases, malformations on ultrasound, alcoholism, and consanguinity. These are well-documented risk factors associated with OCs 1,6,26; however, the correlation of these malformations in OCs with CHD has been minimally explored. Harry 9 reported that no genetic marker or environmental factor was responsible for non-syndromic malformations of both the heart and palate.

There is a scarcity of literature regarding the nature of cardiovascular malformations in populations with OCs 9. In the present study, methodological triangulation of routine cardiological evaluation was performed on a group of individuals with OCs.

In the context of the multidisciplinary program in which this study was developed, a pediatrician evaluated all patients, most of whom had already been subjected to OC correction without a previous cardiological evaluation. Thus, an evaluation of cardiovascular malformations before the surgery, as done by Harry et al. 9, was not possible.

ECG findings of RBBB and AVB in children with OC were also described by Geis et al. 27. These findings are in accordance with the recommendation of Asani et al. 10, who suggested the need for ECG in children with OCs due to the relatively high rate of CHD in this population.

Aside from CHDs, OC patients were also found to have AHDs, with one confirmed case of RHD. Furthermore, there were five cases of MVP (one of which was due to RHD), and 5.2% of cases experienced recurrent tonsillitis. Barbosa et al. 12 described seven MVPs and one bicuspid aortic valve in 24 subjects. MVP and a bicuspid aortic valve, which may be acquired in origin, suggest that patients with OC may be more prone to developing AHD and RF; this increased risk may be related to an increased occurrence of recurrent upper airway infections.

The present study findings identified isolated VSD as the most frequent cardiac malformation, followed by PFO or ASD (isolated or associated with other cardiovascular abnormalities), which agrees with the literature 2,5,9,10. A predominance of mild heart disease was observed in the present study. Severe CHDs cause early death, and without early tracking, they are not diagnosed.

In Brazil, congenital defects have consistently been the second highest cause of perinatal death, contributing to 13% of deaths in the year 2000 28. CHDs are responsible for up to one-third of deaths in neonates with congenital malformations 29. Early diagnosis of CHD and referral to specialized medical centers for treatment are important measures to avoid deaths. It is therefore necessary to improve pre-natal and newborn care, including fetal cardiac evaluation, and utilize the basic health network 30. Amorim et al. 25 emphasized the need for preparation of the health system to diagnose and treat heart diseases earlier, reducing health costs and minimizing the emotional distress of affected patients and their families.

Thus, the present study results suggest an extension of cardiological evaluations that includes not only using echocardiogram in patients with OC but also performing screening by fetal echocardiogram in all patients with an intrauterine diagnosis of OC. This screening may be added to routine clinical, electrocardiographic and echocardiographic evaluations for early tracking of cardiovascular malformations in all patients with OC. Therefore, a line of cardiological care in children with OC that includes the early tracking of cardiovascular alteration by fetal echocardiogram, triangular evaluation at birth (clinical-electrocardiographic-echocardiographic), and cardiological follow-up to evaluate acquired and/or rhythm heart diseases is necessary.

The finding of family risk of developing plurimetabolic syndrome and RHD diagnosis indicates that patients with OC may be more prone to developing AHD. Thus, our findings highlight the importance of anamnesis with attention to personal and family risk factors and methodological triangulation (clinical-electrocardiographic-echocardiographic) in the investigation of patients with OC and the necessity of cardiological follow-up to evaluate acquired and/or rhythm heart diseases. This strategy enables CHD tracking, arrhythmia determination, and AHD prevention and adopts actions to prevent comorbidities and plan individualized treatments. Furthermore, the significant presence of MVP may indicate an increased risk of RHD in individuals with OC, which is a topic that may have to be addressed and evaluated in future studies.

## AUTHOR CONTRIBUTIONS

Rezende AA was the principal investigator and supervised the study. Gilda-Silva-Lopes VL and Rezende AA designed the study. Leite GC was the pediatric cardiologist who evaluated the patients. Leite GC, Costa MI, Freire SS, Maia JM and Brito ME diagnosed the patients. Leite GC, Ururahy MA, Bezerra JF, Lima VM and Luchessi AD recruited the patients and gathered and analyzed the data. Leite GC, Gil-da-Silva-Lopes VL and Rezende AA interpreted the data. Leite GC, Ururahy MA, Gilda-Silva-Lopes VL and Rezende AA wrote and revised the manuscript. All authors have read and approved the final version of the manuscript.

## Figures and Tables

**Table 1:** Descriptive data of patients with oral clefts during clinical evaluation with pediatric cardiologist.

Variables	n=70	%
**Sex**		
Male	42	60.0
**Type of oral cleft**		
Lip/palate cleft	40	57.1
Palate cleft	15	21.4
Lip cleft	15	21.4
**Oral cleft recurrence**		
No	40	57.1
Yes	23	32.9
Unknown*	7	10.0
**Submitted to surgical oral cleft correction**		
Yes	55	78.6
No	11	15.7
Unknown*	4	5.7
**Clinical evaluation by a geneticist**		
Yes	45	64.3
**Syndromic**		
No	36	51.4
Yes	2+	2.9
Suspect	9++	12.9
Unknown*	23	32.9

+ Noonan syndrome (n=1), arthrogryposis (n =1).

++ Suspicious of Marfan (n=1), Goldenhar (n=1), Moebius (n=1), Noonan (n=1), undefined (n=5).

* In these cases, the patients were accompanied by relatives who did not know the answer to the requested information.

**Table 2:** Description of complaints and comorbidities of patients with oral clefts during clinical evaluation with the pediatric cardiologist.

Variables	n	%
**Complaints**	**n=70**	**%**
Yes	28	40.0
**Number of complaints**	**n=28**	**%**
1	13	46.4
>1	15	53.6
**Description of complaints**	n=50*	**%**
Dyspnea on exertion	10	20.0
Cyanosis on exertion	9	18.0
Tachycardia crisis	7	14.0
Cardiac murmur	6	12.0
Chest pain	5	10.0
Recurrent upper airway infections	3	6.0
Syncope	2	4.0
Pain in lower members	2	4.0
Others	6	12.0
**Comorbidities**	**n=70**	**%**
Yes	31	44.3
**Number of comorbidities**	**n=31**	**%**
1	17	54.8
>1	14	45.2
**Description of comorbidities**	**n=58**+	**%**
Recurrent upper airway infections	13	22.4
Short stature	5	8.6
Asthma	5	8.6
Constipation	4	7.0
Recurrent tonsillitis	3	5.2
Seizures	3	5.2
Hyperactivity	3	5.2
Delay in neuropsychomotor development	2	3.4
Hearing deficit	2	3.4
Omphalocele	2	3.4
Others	16	27.6

* The total number of complaints is 50, since some patients presented with more than one complaint at the time of cardiological evaluation.

+ The total number of comorbidities is 58, since some patients presented with more than one comorbidity.

**Table 3:** Description of reported antecedents of patients with oral clefts during clinical evaluation with pediatric cardiologist.

Variables	n	%
**Patients with positive pregnancy antecedents**	39/70	55.7
**Description of pregnancy antecedents**	**n=76**^1^	**%**
Maternal disease	25	32.9
Alcoholism	11	14.5
Maternal age (13 to 15 years old; 36 to 46 years old)	11	14.5
Malformations/abnormalities in obstetric ultrasound	9	11.8
Smoking	7	9.2
Consanguinity	4	5.3
Drug intake (ASA, captopril, valproic acid)	4	5.3
Vaccine for rubella	2	2.6
Exposure to radiation	1	1.3
Illicit drug use	1	1.3
Unknown (adoptive mother)	1	1.3
**Patients with positive neonatal antecedents**	19/70	27.1
**Description of neonatal antecedents**	**n=33**^2^	**%**
Respiratory condition	6	18.2
Neonatal icterus	6	18.2
Cardiac murmur	5	15.2
Prematurity	4	12.2
Low birth weight	3	9.0
Neonatal infection	2	6.0
Heart disease (PDA with Ibuprofen use + PFO)	1	3.0
Others	6	18.2
**Patients with positive family antecedents**	47/70	67.2
**Description of family antecedents**	**n=100**^3^	**%**
Arterial hypertension	20	20.0
Dyslipidemia	17	17.0
Diabetes mellitus	12	12.0
Heart disease	10	10.0
Congenital	5	
Acquired	5	
Syncope	7	7.0
Rheumatic fever	6	6.0
Cardiac murmur	5	5.0
Sudden death (<45 years of age)	4	4.0
Cancer (skin, kidney)	2	2.0
Epilepsy	2	2.0
Syndromes	2	2.0
Early infarction (<47 years of age)	2	2.0
Others	11	11.0
**Patients with positive personal antecedents**	17/70	24.3
**Description of personal antecedents**	**n=23**^4^	**%**
Respiratory	12	52.2
Heart disease	2	8.7
Neurological	2	8.7
Others	7	30.4
**Total**	**23**	**100**

ASA – Acetylsalicylic acid, PDA – patent ductus arteriosus, PFO – patent foramen ovale.

1 25 of 70 (35.7%) patients presented with more than one positive pregnancy antecedent at the time of cardiological evaluation; hence, the total n is 76.

2 19 of 70 (27.1%) patients presented with more than one positive neonatal antecedent at the time of cardiological evaluation; hence, the total n is 33.

3 28 of 70 (40.1%) patients presented with more than one positive family antecedent at the time of cardiological evaluation; hence, the total n is 100.

4 5 of 70 (7.2%) patients presented with more than one positive personal antecedent at the time of cardiological evaluation; hence, the total n is 23.

**Table 4:** Distribution of electrocardiographic findings in patients with oral clefts.

Variables	n	%
**Electrocardiograms performed**	**n=70**	
1 exam	33	47.1
>1 exam	37	52.9
**Evaluation at electrocardiography**	**n=70**	
Normal	54	77.1
Abnormal	16	22.9
**Electrocardiographic abnormalities**	**n=16**	
Right bundle branch block (RBBB)	14	87.6
First degree heart block	1	6.2
RBBB + diffuse alteration of ventricular repolarization*	1	6.2

* Patient diagnosed with arthrogryposis.

**Table 5:** Distribution of echocardiographic findings in patients with oral clefts.

Variables	n	%
**Echocardiograms performed**	**n=70**	
1 exam	60	85.7
>1 exam	10	14.3
**Cardiovascular evaluation at echocardiography**	**n=70**	**%**
Normal	45	64.3
Abnormal	25	35.7
Echocardiographic abnormalities*	**n=25**	**%**
Perimembranous VSD	10	40.0
MVP	5	20.0
PFO	2	8.0
PDA	1	4.0
Dilatation of the ascending aorta +suspected left ventricular noncompaction + suspected endomyocardial fibrosis in right ventricle*	1	4.0
VSD + PFO	1	4.0
PDA + PFO	1	4.0
MVP + PFO	1	4.0
ASD + extrasystoles	1	4.0
ASD + PH	1	4.0
VSD + PFO + PDA	1	4.0
**Total**	25	100
**Severity of heart disease**	**n=25**	**%**
Mild	23	92.0
Moderate (wide ASD + PH)	1	4.0
Severe (suspected left ventricular noncompaction +suspected endomyocardial fibrosis in the right ventricle*)	1	4.0

ASD – atrial septal defect, MVP – mitral valve prolapse, PDA – patent ductus arteriosus, PFO – patent foramen ovale, PH – pulmonary hypertension, VSD – ventricular septal defect.

* Findings in a patient diagnosed with arthrogryposis according to clinical and genetic evaluation. Patient expired before cardiac angiotomography was performed.
